# Emphysematous Cystitis: Report of an Atypical Case

**DOI:** 10.1155/2011/280426

**Published:** 2011-10-23

**Authors:** Karen De Baets, Joost Baert, Luc Coene, Marc Claessens, Robert Hente, Geert Tailly

**Affiliations:** ^1^Department of Surgery, AZ KLINA, 2930 Brasschaat, Belgium; ^2^Department of Urology, AZ KLINA, Augustijnslei 100, 2930 Brasschaat, Belgium

## Abstract

We report the atypical case of a nondiabetic 66-year old male with severe abdominal pain and vomiting who was found to have emphysematous cystitis. Of all gas-forming infections of the urinary tract emphysematous cystitis is the most common and the least severe. The major risk factors are diabetes mellitus and urinary tract obstruction. Most frequent causative pathogens are *Escherichia coli* and *Klebsiella pneumoniae*. The clinical presentation is nonspecific and ranges from asymptomatic urinary tract infection to urosepsis and septic shock. The diagnosis is made by abdominal imaging. Treatment consists of broad-spectrum antibiotics, bladder drainage, and management of the risk factors. Surgery is reserved for severe cases. Overall mortality rate of emphysematous cystitis is 7%. Immediate diagnosis and treatment is necessary because of the rapid progression to bladder necrosis, emphysematous pyelonephritis, urosepsis, and possibly fatal evolution.

## 1. Introduction

Emphysematous cystitis (EC) is a rare but occasionally severe clinical problem. Aetiology is multifactorial and pathogenesis is poorly understood. Most patients diagnosed with EC are elderly diabetic females. Clinical presentation varies individually and may not correlate with the degree of inflammation. Rapid recognition and treatment is necessary to prevent progression to bladder necrosis, emphysematous pyelonephritis (EP), and urosepsis. We present an atypical case of EC in a nondiabetic 66-year old male.

## 2. Case Presentation

A 66-year old schizophrenic male was brought to the emergency department because of severe abdominal pain and vomiting for three hours. He was pale, sweating, and tachypneic. Autoanamnesis was impossible because of his psychiatric comorbidity but heteroanamnesis told us he had not mentioned any other complaints. 

Besides schizophrenia no other pathology was retained in his medical history. His daily medication consisted of an antipsychotic drug, an anticholinergic drug, and a benzodiazepine. 

Physical exam revealed a body temperature of 37.8°C, heart rate of 145 beats per minute, blood pressure of 130/55 mmHg, and oxygen saturation of 91%. Oxygen was administered with a quick increase of the oxygen saturation to 99%. Palpation of the abdomen was extremely painful with diffuse muscular defence. No other abnormalities were found. Physical examination was very difficult since patient was uncooperative.

Laboratory evaluation resulted in a CRP (C-reactive protein) of 49.47 mg/dL (0.00–0.50 mg/dL), white blood cell count of 6.52 × 10³/*μ*L (4.50–11.00 × 10³/*μ*L), prothrombin time of 59% (75–100%), serum creatinine of 6.0 mg/dL (0.70–1.20 mg/dL), BUN (blood urea nitrogen) of 217 mg/dL (8–50 mg/dL), and an eGFR (estimated glomerular filtration rate) of 9 mL/min (>60 mL/min). PSA (prostate specific antigen) measured 141 ng/mL (<6.9 ng/mL). Transaminases were augmented: ASAT (aspartate aminotransferase) 190 U/L (5–34 U/L), ALAT (alanine aminotransferase) 118 U/L (5–41 U/L). Furthermore an LDH (lactate dehydrogenase) of 605 U/L (240–480 U/L) and CK (creatine kinase) of 2206 U/L (<200 U/L) were found. Blood glucose level was 113 mg/dL (70–110 mg/dL).

Macroscopically urine was cloudy. Microscopic urinalysis showed >100 RBC (red blood cell)/HPF (high power field), 50–100 WBC (white blood cell)/HPF and some bacteria. 

**Figure 1:**
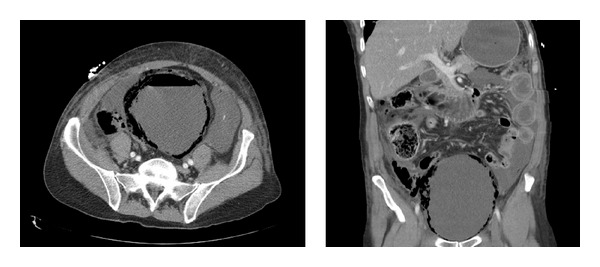
Computed tomography scan of the abdomen showed air in the bladder and the thickened bladder wall, associated with air intra- and retroperitoneally.

Despite painkillers, the patient continued complaining of abdominal pain. Because of the severity of his pain, computed tomography scan of the abdomen was performed. This revealed air in the bladder and the thickened bladder wall, associated with air intra- and retroperitoneally. Furthermore, bilateral pleural fluid, some free fluid in the peritoneal cavity, and a few dilated jejunal segments without characteristics of bowel ischemia were seen (Figures 1(a) and 1(b)). The diagnosis of emphysematous cystitis was made. 

**Figure 2:**
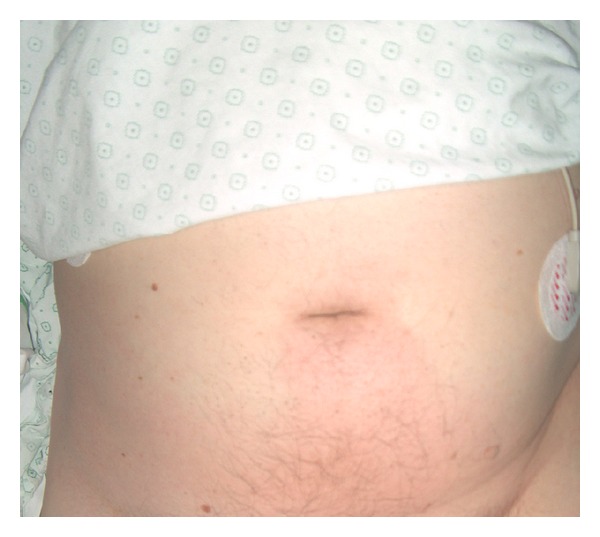
Patient developed a reddishness of the suprapubic region.

Broad-spectrum antibiotics (piperacilline-tazobactam) were empirically started. Intravenous fluid and analgesics were associated. A transurethral catheter was placed and patient was admitted to the surgical department. A few hours later he was transferred to the intensive care unit (ICU) since he became more septic (hypotensive, tachycardiac, tachypneic) and developed a reddishness of the suprapubic region (Figure 2). There was no fever. 

**Figure 3:**
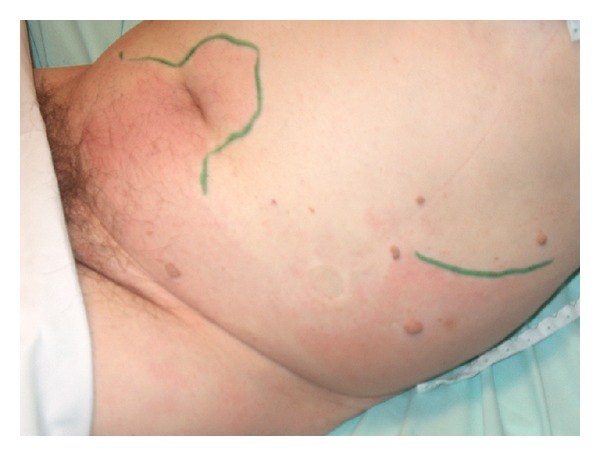
Zone of reddishness expanded up to the flanks bilaterally.

The following days the patient became less septic, renal function improved and the abdominal pain decreased. Contrary to the clinical improvement, the zone of reddishness expanded up to the flanks bilaterally (Figure 3). After two days this reddishness subsided and patient was again admitted to the surgical department. Urine culture revealed more than 10^6^ colonies/mL of *Escherichia coli*. Intravenous piperacilline-tazobactam administration was continued.

**Figure 4:**
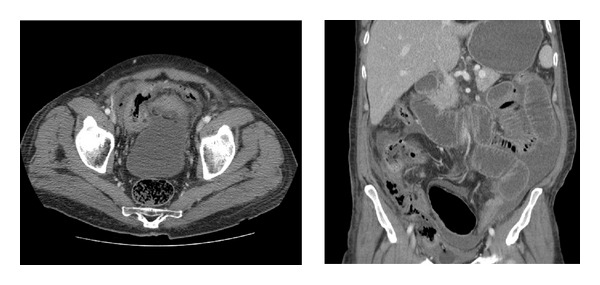
Control computed tomography scan of the abdomen showed necrosis of the bladder wall, predominantly the bladder dome and right bladder wall, with associated peritonitis signs.

Despite continuous intravenous antibiotics and bladder catheterization, patient developed fever (38°C) one week after admission. A control computed tomography scan of the abdomen showed necrosis of the bladder wall, predominantly the bladder dome and right bladder wall, with associated peritonitis signs (Figures 4(a) and 4(b)). The same day an explorative laparotomy was performed. Compatible with the computed tomography scan, the bladder dome and right bladder wall were necrotic and perforated. All necrotic tissue was resected, both ureteral orifices were visualised and preserved. The bladder was closed after placement of a new sterile transurethral catheter. Piperacilline-tazobactam administration was continued per- and postoperative.

A few hours postoperative patient became hemodynamically unstable. Chest X-ray and electrocardiogram were normal. Arterial blood sample showed a haemoglobin level of 4.7 g/dL. Although abdominal drains evacuated no blood a postoperative bleeding was suspected and patient was brought to the operating room for an urgent second look. A venous bleeding of the right iliac vein was visualised and sutured. 

Afterwards patient was sedated, intubated, and ventilated for several days on the ICU. His situation gradually improved and ten days later he was again admitted to the surgical department with the transurethral catheter in situ. Antibiotics were switched to quinolones (levofloxacin). 

**Figure 5:**
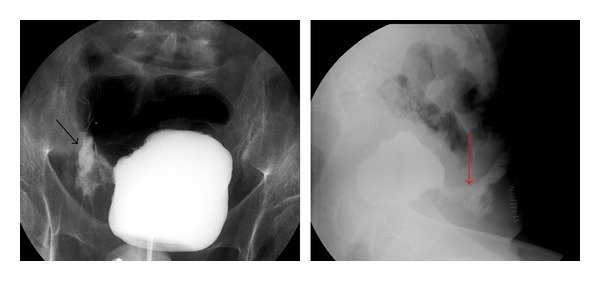
A cystography three weeks postoperatively showed some contrast extravasation into the space of Retzius.

A cystography three weeks postoperatively showed some contrast extravasation into the space of Retzius (Figures 5(a) and 5(b)). Consequently the transurethral catheter was left in situ. 

Levofloxacin administration was ceased after a sterile urine culture 25 days postoperatively.

**Figure 6:**
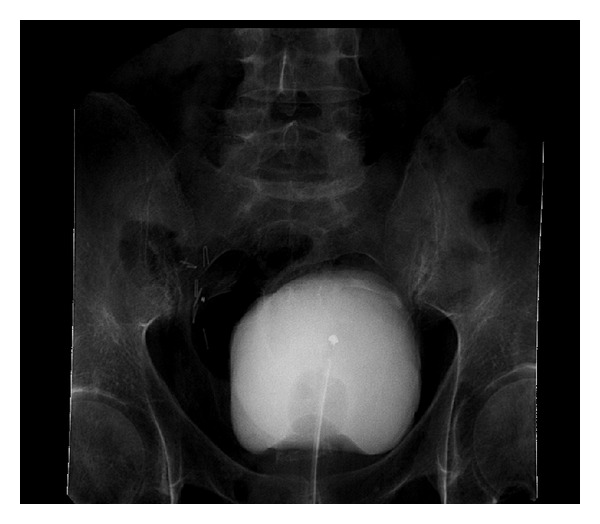
A control cystography six weeks postoperatively revealed no contrast leakage anymore.

Two weeks later patient left the hospital with the transurethral catheter in situ, which he himself promptly removed on the third day following his discharge. Afterwards no voiding problems were seen so no new drainage was provided. A control cystography one week later revealed no contrast leakage anymore (Figure 6). Patient was comfortable, voiding was uncomplicated with good voiding volumes and no residual volume, and patient was again discharged.

Pathology report of the partial cystectomy specimen showed only necrotic tissue, Gram- and PAS staining (periodic acid-Schiff) were negative.

## 3. Discussion

EC is an infection of the bladder associated with gas production, usually occurring in elderly diabetic females. Thomas et al. [1] identified that two-thirds of all reported cases of EC until 2006 were diabetic and 64% were women, with a median patient age of 66 years. In the series of Grupper et al. [2] the same percentages were found. Kuo et al. [3] also found a predilection of EC for women, according to them due to an increased susceptibility of women to urinary tract infections. In contrast to these findings we presented the atypical case of a 66-year old nondiabetic male.

The aetiology and pathogenesis of EC remain poorly understood. In diabetic patients an elevated tissue and urinary glucose level is a plausible source of fermentation for gas-forming bacteria. This process is possibly favoured by associated impaired renal perfusion, diabetic nephropathy, bladder dysfunction secondary to neuropathy, and impaired leukocyte function. In non-diabetic patients higher levels of urinary albumin, lactose, or tissue proteins can result in the formation of H_2_ (hydrogen) and CO_2_ (carbon dioxide) gas. In all cases it concerns an inappropriate host response to the causative microorganism. Urinary tract obstruction, and consequently urinary stasis, is another major risk factor besides diabetes. All patients with recurrent urinary tract infections, indwelling urethral catheter, neurogenic bladder, immunosuppressive comorbidity, and others. are predisposed to complicated UTIs (urinary tract infection) such as EC. Grupper et al. [2] found an incidence of malignancy of 8% associated with EC in their series, Kuo et al. [3] found an association with malignancy in 16.7%. No other reports of malignancies associated with EC were found.

Clinical features of EC are nonspecific and vary individually. Some patients are asymptomatic or report only minor voiding problems or abdominal pain, while others present with septic shock. Thomas et al. [1] found that 7% of the reported cases in literature were asymptomatic and diagnosed incidentally by abdominal imaging for other concurrent conditions. A recent study of Kuo et al. [3] showed no significant clinical manifestation suggesting the presence of EC. Furthermore clinical presentation does not correlate with the severity of inflammation. Grupper et al. [2] reported that classic symptoms of UTI were seen in only approximately 50% of the cases. The most common symptom in their series was abdominal pain. EC is a possibly life-threatening disease because of rapid progression to bladder necrosis, EP, urosepsis, and death. To avoid these complications prompt evaluation and treatment is necessary. 

Since clinical presentation of EC is very different and nonspecific, EC is diagnosed radiologically. Most accurate examination is the computed tomography scan of the abdomen/pelvis [1–3]. Because of the increased use of computed tomography scan, a greater incidence of emphysematous infections is seen. Other important factors for this are a greater awareness for such pathology and an increase in number of diabetic and elderly patients. As not all patients with symptoms of urinary tract infection are submitted to abdominal imaging, the number of cases may be underestimated. Thomas et al. [1] found that computed tomography scan was used in 40% of the cases of EC until 2006. Simple plain film of the abdomen was the most common imaging method, used in 84%. An abdominal X-ray has a very low specificity however. Kuo et al. [3] found that simple radiograph of the abdomen was diagnostic in only 13%. Computed tomography scan also differentiates other pathology associated with air in the urinary tract (i.e., uro-intestinal fistulae, renal infarction, trauma, instrumentation). Moreover the presence of EP can be evaluated. Computed tomography scan in our case showed air in the bladder lumen, the bladder wall, the peritoneal cavity, and the retroperitoneal space. The combination of presence of air in all these compartments is by our knowledge never been reported in the literature before. Abdominal ultrasound and MRI (magnetic resonance imaging) are less valuable as imaging modalities because of difficult interpretation. Cystoscopy alone is not sufficient to diagnose EC but it can evaluate the presence of bladder outlet obstruction. A pathological assessment typically reveals a thickened bladder wall with multiple gas-filled vesicles, predominantly in the bladder mucosa, surrounded by flattened fibrocytes and multinucleated giant cells [4, 5]. 

Multiple gas-producing microorganisms can cause EC. *Escherichia coli, Klebsiella pneumonia, Enterococcus, Candida, Clostridium perfringens*, and many others were all isolated in urinary culture, with *Escherichia coli* being the most prevalent followed by *Klebsiella pneumonia* [1–3]. The same pathogens were found in cases of EP [3, 6, 7]. Most organisms are facultative anaerobic, only in rare cases a pure anaerobic isolate, multiple organisms, or no organisms are found.

The management of EC generally consists of broad-spectrum antibiotics, bladder drainage, correction of the blood glucose level, and treatment of any underlying comorbid disorders. In most cases broad-spectrum antibiotics are started. Once the sensitivities of the isolated urinary pathogens are known, antibiotics should be switched to more specific ones. No consensus exists about the duration of antibiotic treatment. Grupper et al. [2] found a median length of ten days, the median length of hospital stay was seven days. In severe cases, or if the patient does not respond to conservative treatment, surgery is needed (partial cystectomy, cystectomy, surgical debridement). In the series of Thomas et al. 10% [1] of the patients underwent surgery. In the series of Grupper et al. [2] 15% needed a laparotomy. This group needing surgery was not significantly different from the nonsurgical group for demographic or comorbidity parameters. Postoperatively, antibiotic treatment, bladder drainage and control of blood glucose level, and other risk factors should be continued.

Thomas et al. [1] published an overall mortality rate of EC of 7%. In the series of Grupper et al. [2] a mortality rate of 9.4% was found. This indicates that EC can be successfully treated with conservative management alone and is not as severe as feared. When another emphysematous infection of the urinary tract is associated the mortality rate increases to14% [1]. No other significant predictors of poor outcome were identified.

## 4. Conclusions

Emphysematous cystitis is an uncommon infectious condition of the bladder caused by gas-producing micro-organisms Because of the possible insidious clinical presentation it is of utmost importance that emergency physicians are aware of this clinical entity, especially in high risk patients. Immediate diagnosis and treatment is necessary because of the rapid progression to bladder necrosis, EP, urosepsis, and possibly fatal evolution.

## Abbreviations

EC: Emphysematous cystitis
EP: Emphysematous pyelonephritis
CRP: C-reactive protein
BUN: Blood urea nitrogen
eGFR: Estimated glomerular filtration rate
PSA: Prostate specific antigen
ASAT: Aspartate aminotransferase
ALAT: Alanine aminotransferase
LDH: Lactate dehydrogenase
RBC: Red blood cell
HPF: High power field
WBC: White blood cell
ICU: Intensive care unit
PAS: Periodic acid-Schiff
H_2_: Hydrogen gas
CO_2_: Carbon dioxide gas
UTI: Urinary tract infection
CT: Computed tomography
MRI: Magnetic resonance imaging.
